# Patients with HPV‐associated oropharyngeal head and neck cancer have higher rates of weight loss and increased supportive needs

**DOI:** 10.1002/jmrs.360

**Published:** 2019-12-19

**Authors:** Teresa E. Brown

**Affiliations:** ^1^ Royal Brisbane & Women's Hospital Brisbane Queensland Australia; ^2^ School of Human Movement & Nutrition Sciences University of Queensland St Lucia Queensland Australia

## Abstract

This editorial discusses the new evidence coming to light that patients with HPV‐associated head and neck cancer are at increased risk of nutritional decline during treatment, with increased weight loss and higher need for tube feeding. Due to the anticipated epidemic of this particular disease, there is an ongoing need for research to ensure nutrition care outcomes and supportive care needs are optimised in this sub group, which will improve clinical outcomes and enhance patient quality of life.

This present issue of the Journal of Medical Radiation Sciences features a study by Anderson et al. that reports findings on patients with HPV‐associated head and neck cancer. This subgroup experience increased weight loss compared to other groups of patients who are typically considered a higher nutrition risk.^1^ Their study highlights the changing landscape of head and neck cancer radiation therapy patients and that patients with HPV‐associated disease may require additional support and interventions to optimise their nutrition outcomes.

The risks of malnutrition, weight loss and dysphagia are well known in this patient population with many patients requiring enteral nutrition. There is a considerable body of evidence to support that malnutrition and weight loss have adverse consequences. This includes the impact of malnutrition on the patient's well‐being and quality of life, as well as increasing risk of complications, treatment interruptions, unplanned hospital admissions and increased length of stay, thus contributing to increased costs to the health service system. Weight loss has also been found to be a major prognostic indicator for survival. Patients who presented with >10% weight loss pre‐treatment had worse overall survival and disease‐specific survival. In addition, patients who experienced critical weight loss during radiation therapy also had worse disease‐specific survival.^2^ As discussed by Anderson et al., the evolution of highly conformal radiation therapy techniques has allowed high dose escalation to tumour volumes whilst sparing dose volumes to surrounding organs at risk and healthy tissue. Maintaining weight during treatment is thus essential to avoid treatment‐induced anatomical changes. Such changes can result in potential underdosing and/or overdosing of target volumes and organs at risk volumes. This can subsequently impact upon resource utilisation to perform time‐intensive adaptive radiation therapy and re‐planning. Therefore, optimal nutrition care plays a significant role in optimising patient outcomes.

The study by Anderson et al. investigated whether a patient risk stratification model to predict patients most likely to benefit from prophylactic gastrostomy placement correlated with reported weight loss during radiation therapy. Weight‐loss outcomes were reported according to their risk stratification level: high risk (T3 or T4 with level 2 lymphadenopathy), high‐intermediate risk (T3 or T4 without level 2 lymphadenopathy) and low‐intermediate risk groups (T0, T1 or T2 with level 2 lymphadenopathy). Overall, there was good adherence (87%) to their prophylactic feeding tube recommendations. The weight‐loss outcomes were similar for those with a feeding tube placed (*n *=* *87) compared to the whole cohort (*n *=* *101). Interestingly, the low‐intermediate risk group lost significantly more weight than the high risk and high‐intermediate risk groups; 8.2% vs 4.8% and 5.2%, respectively. Furthermore, the low‐intermediate risk group had high rates of feeding tube placement (*n *=* *36 received a tube out of the 42 recommended in this group) and a lower disease burden. However, this group was characterised by a significantly higher proportion of patients with HPV‐associated disease, with the typical patient characteristics associated with this disease profile, such as younger age, healthy BMI, no pre‐existing weight loss/dysphagia/tumour burden, no comorbidities, non‐smoker and good performance status. Similarly, Vangelov et al. found patients with HPV‐associated disease had increased weight loss during treatment, greater incidence of critical weight loss (≥5%) and were more likely to require a feeding tube.^3^

Anderson et al. attributes the poor nutritional outcomes for patients with HPV‐associated disease to a lack of adherence to nutrition recommendations given, and thus sub‐optimal feeding tube utilisation. A recent randomised controlled trial observed poor patient adherence to recommended feeding tube use, thus supporting Anderson et al. theory.^4^ The study cohort represented patients with head and neck cancer considered high nutrition risk who received a prophylactic gastrostomy as part of their treatment (*n *=* *125) and consisted predominantly of patients with oropharyngeal cancer (78%) and p16‐positive disease (71%). Patients were randomised to receive early supplementary nutrition support via the gastrostomy tube before the start of treatment versus commencement of nutrition support when it became clinically indicated during treatment. The authors found that there was only 51% adherence to this early phase of feeding in the intervention arm. Adherence to the clinical phase of tube feeding, when it became clinically indicated during treatment, was higher in the intervention group at 58% vs 38% in the standard care group. However, these adherence rates were still deemed low overall and a likely explanation for the ongoing weight loss seen in this patient population.

Patient‐reported barriers to nutrition care and enteral feeding recommendations in this study were broad and included uncontrolled nutrition impact symptoms (e.g. nausea, reflux), psychosocial patient factors (e.g. mood, finances) and environmental factors (e.g. hospital environment/appointments and lack of time). This highlights that patients may have unmet supportive needs either in relation to their symptom management and/or adequate information from their dietitian or other health professionals to address their knowledge or other psychosocial factors which may improve their engagement with nutrition recommendations. Utilising behaviour change interventions, such as cognitive behavioural therapy and motivational interviewing, have been shown to be an effective strategy to improve adherence and nutrition outcomes. A recent multicentre Australian study has demonstrated that these approaches can be effectively used by trained dietitians as part of their usual dietary counselling consultations for patients with head and neck cancer, resulting in improved nutrition outcomes and quality of life, as well as reducing depression scores and treatment interruptions.^5^

Other possible explanations for poorer nutrition outcomes in this patient subgroup could be due to the increased acute toxicities experienced during treatment. Becker‐Schiebe et al. reported this phenomenon, finding p16‐positive tumours to have increased acute toxicities compared to p16‐negative tumours. Their study demonstrated significantly higher incidences of grade 3 radiodermatitis, mucositis and dysphagia.^6^ In addition, as these patients present with minimal symptoms at diagnosis, the impact of the acute toxicities experienced during treatment is likely to cause increased distress with a greater impact on their well‐being and larger decline in their quality of life compared to patients who present with HPV‐negative disease who are already experiencing symptoms of tumour burden at the time of diagnosis. Thus, it is anticipated patients with HPV‐associated disease are likely to have an increased need for psychological support to help them cope with these dramatic changes.

There has been much debate in the literature regarding the optimal treatment for patients with HPV‐associated disease. As these patients are generally younger and have a better prognosis, they are living with late treatment toxicity for a longer period resulting in a significant impact on patient quality of life and survivorship. This justifies the abundance of research currently investigating de‐intensification of treatment in order to reduce toxicities without compromising on disease or survival outcomes. Deschuymer et al. recently stated numerous trials investigating changes to primary concomitant systemic therapies, reduced radiation therapy doses and radiation therapy dose adaptation after induction chemotherapy.^7^ Additionally, there is a resurgence towards primary surgery using minimally invasive techniques. Transoral laser surgery and transoral robotic surgery have more acceptable functional outcomes compared to historical open surgery.^7^ There are also other emerging treatments, such as immunotherapy and proton therapy, which require consideration in regard to their acute and chronic toxicities and their impact on patient outcomes.^7^ It is anticipated that the optimal treatment for HPV‐positive oropharyngeal cancer may well not be known for another decade as the outcomes from these trials are reported. However, preliminary findings from the RTOG 1016 trial have reported disappointing findings. The de‐escalation study compares concurrent chemoradiation therapy with cetuximab instead of cisplatin, with inferior overall survival and progression‐free survival and no benefit with reduced toxicities.^8^ Thus, it is still likely to see patients with high levels of acute and long‐term toxicities for some time, as radiation therapy plus cisplatin remains the current recommended standard care until evidence can suggest otherwise.

The rising prevalence of HPV‐associated oropharyngeal cancers is expected to continue. Until the impacts of vaccination programs are realised, current supportive care management strategies require optimisation and adaptation to meet the needs of this unique subset of patients with head and neck cancer. Current risk stratification tools or clinical pathways to determine nutrition risk may be over‐simplistic and have limited clinical utility to identify this unique subgroup of patients. Therefore, further development and refinements to existing tools are necessary to ensure they meet the needs of this specific patient population who have been found to be particularly vulnerable to nutrition decline. Further qualitative research will be beneficial to fully understand the barriers that patients experience to nutrition care and enteral feeding so that strategies can be developed to address these gaps. Consumer engagement to assist with the co‐design of future models and systems of supportive care are also important to ensure they are patient‐centred and provide timely access to care when required, such as the use of patient‐reported outcome measures and/or routine comprehensive screening for allied health services.

## Conflict of interest

No conflicts of interest to declare.
